# The Vitamin D Role in Preventing Primary Headache in Adult and Pediatric Population

**DOI:** 10.3390/jcm10245983

**Published:** 2021-12-20

**Authors:** Giovanni Battista Dell’Isola, Eleonora Tulli, Rossella Sica, Valerio Vinti, Elisabetta Mencaroni, Giuseppe Di Cara, Pasquale Striano, Alberto Verrotti

**Affiliations:** 1 Department of Pediatrics, University of Perugia, Giorgio Menghini Square, 06129 Perugia, Italy; eleonora.tulli26@gmail.com (E.T.); rossellasica@libero.it (R.S.); vinti.valerio@gmail.com (V.V.); elisabetta.mencaroni@ospedale.perugia.it (E.M.); giuseppe.dicara@unipg.it (G.D.C.); alberto.verrottidipianella@unipg.it (A.V.); 2 Pediatric Neurology and Muscular Diseases Unit, IRCCS “G. Gaslini” Institute, Gerolamo Gaslini Street, 5, 16147 Genoa, Italy; pstriano@unige.it; 3 Department of Neurosciences, Rehabilitation, Ophthalmology, Genetics, Maternal and Child Health, University of Genoa, Paolo Daneo Square, 3, 16132 Genoa, Italy

**Keywords:** headache, migraine, vitamin D, pharmacological prophylaxis, nutraceutical

## Abstract

Headache is among the main neurological disorders with a great impact on both adults and children. The diagnosis of primary headache and proper management is often delayed with a great impact on work productivity and overall quality of life. Chronic headache often requires prophylactic therapy to reduce the frequency and severity of the attacks and the use of abortive medications. Besides the use of several classes of drugs, another treatment modality is the use of Nutraceuticals. Some studies have suggested a possible role of vitamin D in headache prophylaxis. Indeed, vitamin D is involved in several pathways of brain development, neuroprotection and neurotransmission. Moreover, there is data suggesting a close relationship between primary headache and vitamin D deficiency, both in children and in adults. To date, a few studies have evaluated the effect of vitamin D on headaches. The aim of this review is to summarize the data collected on headache prophylaxis with vitamin D comparing the effects of vitamin D in pediatric and adult populations.

## 1. Introduction

Headache is a very common disorder in both adults and children with a great impact on their lives and that of family caregivers. It is among the main neurological disorder with a prevalence of almost 50% in the general population [1,2]. Headache prevalence rises with increasing age, with a peak between 35 and 39 years old. Females are more affected than males, regardless of ethnicity [3].

Headaches can be primary or recognize underlying pathological conditions. Primary headaches constitute almost 98% of all headaches and are not life-threatening. The main types of primary headache, as reported in the International Classification of Headache Disorders (ICHD-3 beta) [4], are muscle tension headache (TTH), migraine and cluster headache (CH). Secondary headaches are uncommon, but an early diagnosis is essential to undertake a life-saving intervention. The most common causes of secondary headaches are viral and bacterial infections (especially infections of the upper respiratory tract), sinusitis, intracranial space-occupying lesion and traumas.

The diagnosis of primary headache and proper management are often delayed with a great impact on work productivity and overall quality of life [5]. A healthy lifestyle can prevent and limit headache episodes [6,7]. However, chronic headache often requires prophylactic therapy to reduce the frequency and severity of the attacks. Besides the use of several classes of drugs, another treatment modality is the use of Nutraceuticals (a food or part of food useful in the prevention and/or treatment of disease) [8]. Some studies have suggested a possible role of vitamin D in headache prophylaxis. 

Vitamin D is a steroid hormone that exists in two major forms: vitamin D2, or ergosterol and vitamin D3, produced in the skin from 7-dehydrocholesterol [9,10]. Vitamin D3 is then metabolized by 1-alpha-hydroxylase to 1,25-(OH)2 D (or calcitriol). Calcitriol binds vitamin D receptor (VDR), a nuclear receptor that controls several genes involved in metabolism, cells adhesion, cells proliferation, tissue differentiation, angiogenesis, inflammation and epigenetic [9]. VDR has been detected in almost every cell and tissue not only in the intestine, bone, cartilage and kidney but also in the heart, immune system and central nervous system [10]. Indeed, several extra-skeletal effects of vitamin D have been revealed in the last decades, including diminishing inflammation, influencing the immune systems, preventing cancer, controlling cardiovascular disorders, endocrine abnormalities and neurological and psychological diseases [11,12,13].

In this topical review, we aim to summarize the data collected on headache prophylaxis with vitamin D comparing the effects of vitamin D in pediatric and adult populations.

## 2. Vitamin D and Brain

In recent years, considerable attention has been given to the pathogenic role of Vitamin D in neurodegenerative, neuroinflammatory and neuropsychological diseases such as headache, cognitive decline, Alzheimer’s diseases, depression, psychosis and autism [14,15,16]. Vitamin D is a well-recognized neurosteroid that plays an important role in both embryonic and adult brains [17,18,19,20], albeit the mechanism by which this occurs is not completely known. 

Vitamin D plays interactions with nuclear receptors (VDR) and surface receptors known as protein disulfide isomers family A members 3 (PDIA3) resulting in genomic and non-genomic actions. Vitamin D binding with VDR influences genomic transcription, with the enhancement of calbindin, cathelicidin, transforming growth factor and nerve growth factor and inhibition of parathyroid hormone and 1-alpha-hydroxylase transcription [21]. Both nuclear and surface receptors induce non-genomic actions such as regulation of Ca^2+^ transport and adjustment of activity of adenylyl cyclase, phospholipase C, protein kinase C and p38 MAP kinase [18]. The expression of VDR in different brain regions represents indirect evidence of vitamin D activity. Indeed, the extensive expression in the neuroepithelium suggests that vitamin D is involved in the proliferation and differentiation of neuronal stem cells [22,23]. In addition, the expression of VDR in the hippocampus and the limbic system indicates the role of vitamin D in memory and cognitive function [24,25]. VDR is also present in the sensory cortex suggesting an involvement in the somatosensory system and in the substantia nigra confirming the link between vitamin D and dopamine [26,27,28]. Although the classic metabolism of vitamin D is conducted by the liver and kidneys, there is evidence that vitamin D is also synthesized and metabolized in the brain [18]. Vitamin D plays a paracrine and autocrine action in proliferation, differentiation and cell survival. Vitamin D deficiency may lead to increased cellular proliferation with defective differentiation [22,29]. Alterations in brain structure at birth such as larger lateral ventricles and thinner cortex were documented in animal models with maternal hypovitaminosis D [23,30]. Vitamin D is also implicated in dopamine metabolism by influencing the expression of specification factors for dopaminergic phenotypes such as Nurr1 and P57kip2 [31]. Therefore, vitamin D deficiency alters dopamine signaling and related processes such as motivation, reward, addiction behavior and motor function [24,32]. Moreover, vitamin D modifies serotonin neurotransmission through genomic regulation of tryptophan hydroxylase 2 (TH2), a key enzyme in serotonin production [33].

In conclusion, vitamin D is involved in several pathways of brain development, neuroprotection and neurotransmission. A better understanding of these processes could aid in the development of replacement therapy with vitamin D in neurological disorders. 

## 3. Vitamin D and Headache 

Several studies demonstrated a close relationship between primary headache and vitamin D deficiency, both in children [34,35,36] and in adults [37,38,39]. This association is also confirmed by the increased prevalence of headaches in autumn and winter at high latitudes, where vitamin D levels are generally low [40]. Vitamin D and headache are linked by multiple and complex mechanisms involving headache pathophysiology [41]. 

Migraine attacks start with the activation of meningeal nociceptors at the origin of the trigeminovascular pathway [41,42,43]. The consequent release of vasoactive neuropeptides, as calcitonin gene-related peptide (CGRP), substance P, neurokinin A and nitric oxide (NO), leads to intracranial blood vessels dilation, plasma proteins extravasation, platelets aggregation and mast cells degranulation. This neurogenic inflammation generates the nociceptive signal, that from the spinal trigeminal nucleus is processed to the thalamus and to cortical structures. [44,45,46,47]. In addition, the activation threshold of peripheral trigeminovascular neurons decreases after stimulation [42,47]. Oxidative stress is an important trigger of the trigeminovascular pathway, and it causes the maintenance of migraine [48]. Glutamate and serotonin are also involved in migraine pathophysiology. Indeed, increased levels of glutamate play a key role in pain transmission, central sensitization and cortical spreading depression [49]. Serotonin decreased level leads to neuropeptides release from the trigeminal nerves [50]. The hypothalamus plays a key role in pain modulation, as proved by its anatomical connections with trigeminovascular neurons and by migraine accompanying symptoms, such as changes in appetite, sleep-waking rhythms alterations, hypersensitivity to stimuli and mood changes [51]. 

In TTH, both peripheral mechanisms (nociception from pericranial and cervical muscles) and central factors (central sensitivity) participate in the development of pain [52,53,54,55]. Some studies suggest that also NO is involved in TTH pathophysiology [56]. 

In CH pain is generated by the activation of the peripheral trigeminal component, with consequent cerebral vascular changes caused by trigeminal-autonomic reflex activation. The hypothalamus plays a key role, as demonstrated by the circannual and circadian rhythmicity of the attacks [52,57]. 

**Figure 1:**
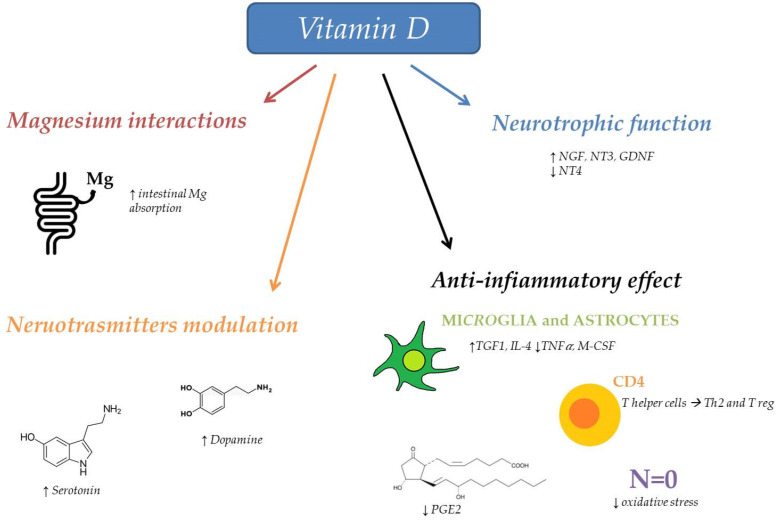
Vitamin D role in headache prevention. Abbreviation: Magnesium (Mg), neural growth factor (NGF), nuerotrophin 3 (NT3), glial cell line derived neurotrophic factor (GDNF), neurotrophin 4 (NT4), transforming growth factor beta 1 (TGF1), interleukin-4 (IL-4), tumor necrosis factor-*α* (*TNF α*), macrophage colony stimulating factor (*M-CSF*).

Vitamin D can contrast the trigger and maintenance of primary headaches through different pathways (Figure 1). Both the initiation and continuation of headache are opposed by the anti-inflammatory effect of vitamin D, which prevents the neuroinflammation underlying migraine, musculoskeletal pain and TTH [10,11,58]. Indeed, vitamin D is able to shift the balance of T helper cells to Th2 and regulatory T cells [59], to inhibit prostaglandin E2 synthesis [60], to contrast NO-synthase and thus to reduce NO production. Vitamin D involvement in inflammatory pathways is also mediated by its effect on non-neuronal cells. Indeed, vitamin D upregulates transforming growth factor beta 1 and interleukin-4 and suppresses tumor necrosis factor-α and macrophage colony stimulating factor in astrocytes and microglia [61]. The anti-inflammatory effect of Vitamin D is confirmed by the inverse association between the C-reactive protein and vitamin D levels [62]. 

Furthermore, vitamin D reduces oxidative stress, another migraine trigger, which contributes also to its maintenance. Vitamin D modulates the rate of oxygen consumption [11,63] and controls glutathione metabolism, which helps to eliminate nitrogen and oxygen reactive species in astrocytes [11].

Another mechanism underlying the protective effect of vitamin D in both migraine initiation and maintenance involves the direct relationship between vitamin D and magnesium (Mg) serum concentrations. Indeed, vitamin D promotes intestinal absorption of Mg, which has been demonstrated to be effective against chronic pain conditions and migraine by contrasting vascular and neurogenic mechanisms [47,64].

Moreover, vitamin D opposes the maintenance of headaches by influencing dopamine and serotonin synthesis via tyrosine hydroxylase. Indeed, both dopamine and serotonin are neurotransmitters connected with the pathogenesis of migraine [65]. By increasing serotonin levels, vitamin D could also be useful in the control of depressive symptoms, often associated with headaches [10,66]. 

In addition, vitamin D can influence the modulation of pain regulating the synthesis of neurotrophic factors including neural growth factors [65]. 

The connection between vitamin D and primary headache is also confirmed by the presence of VDR in the hypothalamus, a central structure for the modulation of pain [40]. In particular, cross-sectional research by Mottaghi et al. showed a link between migraine and two gene polymorphisms of VDR. The TaqI and FokI VDR polymorphisms were associated with migraine without aura. FokI heterozygosis was related to headache severity [10,38].

## 4. Vitamin D Prophylaxis in Adult Headache

According to data published so far, vitamin D supplementation may play a role in headache treatment, with a focus on the prevention of headache attacks. To date, most clinical trials in the literature of vitamin D supplementation in headache patients showed that it may be beneficial in some patients, especially in those with vitamin D deficiency.

We identified studies investigating the effect of vitamin D supplementation on various headache parameters such as duration, frequency and severity (Table 1). Many studies revealed that vitamin D supplementation had an impact on reducing headache frequency, especially for migraine.

Several underlying mechanisms have been suggested to play a role in migraine pathogenesis with neuroinflammation having an emerging role. Therefore, it is suggested that using anti-inflammatory agents as adjuvants together with prophylactic drugs in the pharmacologic management of migraine might be effective. The anti-inflammatory effects of vitamin D3 are known. In a randomized, double-blinded, placebo-controlled, parallel trial of vitamin D3 supplementation in adult patients with migraine, Gazerani et al. reported a significant reduction in the frequency of migraine attacks over time in patients treated with D3-vitamin. However, migraine-related symptoms were not significantly affected by the supplementation [67]. Similarly, Mottaghi et al. in a randomized, double-blind placebo-controlled trial of vitamin D3 supplementation reported a reduction in the frequency of migraine in the treatment group compared to controls and a significantly lower mean headache diary record. Besides, no modification in severity and duration of migraine attacks was reported [62]. In a randomized, placebo-controlled study, the supplementation of vitamin D was administered together with simvastatin. The treatment group was given simvastatin 20 mg twice-daily plus vitamin D 1000 international units twice-daily for the prevention of episodic migraine. The results showed a reduction in the number of days with migraine and the use of abortive medications [68]. A placebo-controlled, double-blind study by Ghorbani et al. investigated the effects of vitamin D3 supplementation on headache characteristics and serum levels of pro/anti-inflammatory markers in migraineurs. The vitamin D3 supplemented group experienced significantly lower headache days per month, reduced attacks duration, less severe headaches, and lower analgesics use/month than the placebo group [69]. In two posthoc analyses, the same authors evaluated the effects of vitamin D3 supplementation respectively on interictal serum levels CGRP in episodic migraine patients and on TGF-β and IL-17 serum levels in migraineurs. There is evidence in the literature that CGRP levels might be augmented in migraineurs especially during head pain periods. CGRP plays a role in vasodilation, modulating the immune system, neuronal inflammation and transmission of pain signals [70]. The first posthoc analyses showed that vitamin D3 supplementation in episodic migraine might potentially improve headache characteristics and disability probably through attenuating CGRP levels [71]. In the second posthoc analyses, the intervention group had a higher TGF-β serum level with vitamin D3 supplementation, also preventing the increment in IL-17 serum level revealing that modulation of Th17/Treg cell balance could be helpful in the improvement of migraine related pathways [72]. Ylmaz et al. administered 50,000 IU/week vitamin D3 in patients with chronic widespread musculoskeletal pain and headache reporting an improvement in symptoms severity and quality of life [73]. Conversely, a randomized, double-blind, placebo-controlled trial among adult ethnic minorities in Norway investigating the effect of vitamin D on musculoskeletal pain and headache showed no significant effect on the occurrence, anatomical localization, and degree of pain or headache compared to placebo. Lacks and weaknesses of this trial which are to mention are the focus on symptoms in a presumed healthy ethnic minority population, excluding defined illnesses. Moreover, the authors did not differentiate between the different types of headaches and low doses of daily vitamin D3 supplementation were used in this trial (respectively vitamin D3 25 µg/d, vitamin D3 10 µg/d, or placebo in the study groups) [74]. A recent randomized controlled trial by Rist et al. evaluated whether vitamin D or marine-3 fatty acid supplementation may reduce migraine frequency or severity in middle aged or older adults. The trial showed no changes in migraine frequency or severity. An important limitation of this study is that the authors did not quantitatively measure migraine frequency or severity and all changes in migraine frequency and severity are based on self-report [75].

It is known that cluster headache shows a seasonal predilection, with nocturnal attacks [76]. It is suggested that vitamin D might have a role in CH as diurnal and seasonal variation might be related to sunlight and vitamin D metabolism. In a preliminary study, Sohn et al., reported that vitamin D deficiency is common in patients with cluster headaches, but the role of vitamin D deficiency is uncertain, except for its seasonal influence [77]. To our knowledge, only a poster presentation showed that vitamin D supplementation could be effective in preventing CH attacks. Batcheller, indeed, presented survey results of 110 CH sufferers using a daily anti-inflammatory regimen of vitamin and mineral supplements including 10,000 IU/d vitamin D3 and Omega-3 fish oil as a CH preventative. 80% of CH patients reported significant reductions in frequency, duration and severity of headache [78]. A clinical trial is currently underway to assess the role of high dose vitamin D plus a multivitamin in the prevention of CH [79].

To our knowledge, there is currently a lack of data in the literature about the relationship between other types of primary headache and vitamin D.

No major adverse events have been reported in the literature in vitamin D supplementation, making it a safe form of treatment. Despite the promising results of the studies reported in this review, further studies are needed to assess the role of vitamin D supplementation in headache management and prophylaxis.

**Table 1:** Vitamin D Prophylaxis in Adult Headache.

Reference	Study Design	Sample Size (Mean Age)	Type of Headache	Dose of Vitamin D Supplementation	Treatment Duration	Results
Gazerani (2019) [67]	Randomized, double-blind, placebo-controlled, parallel	48 (45.5 y)	Migraine	100 μg/day	196 days	Decreased migraine frequency, no effect on severity, pressure pain thresholds, or temporal summation.
Mottaghi (2015) [62]	Randomized, double-blind placebo-controlled	65 (Group 1 32.7 ± 10.6 y; Group 2 33.9 ± 11.6 y)	Migraine	50,000 IU/week	10 weeks	Decreased headache frequency and mean headache diary results, no effect on severity and duration of headache.
Buettner (2015) [68]	Randomized, placebo-controlled	57 (40 y)	Episodic migraine	1000 IU twice per day (+ simvastatin 20 mg/twice per day)	24 weeks	Decreased number of migraine days
Ghorbani (2020) [69]	Randomized double-blind placebo-controlled trial	80 (Group1 37 y; Group 2 38 y)	Episodic migraine	2000 IU/day	12 weeks	Significantly lower headache days per month, reduced attacks duration, less severe headaches and lower analgesics use/month
Ghorbani (2020) [71]	Randomized double-blind placebo-controlled trial	80 (Group1 37 y; Group 2 38 y)	Episodic migraine	2000 IU/day	16 weeks	CGRP level appeared to be significantly lower following vitamin D supplementation. Improved headache characteristics and disability
Ghorbani (2021) [72]	Randomized double-blind placebo-controlled trial	80 (Group1 37 y; Group 2 38 y)	Episodic migraine	2000 IU/day	12 weeks	Enhanced Th17/Treg related cytokines balance in episodic migraineurs.
Yilmaz (2016) [73]	Pre-post	29 (36.9 y)	Headache	50,000 IU/weekly + calcium of 1000 mg/day	3 months	Decreased headache severity and frequency.
Knutsen (2014) [74]	Randomized double-blinded placebo-controlled parallel-group	158 (35–40 y)	Headache	Group 1: 25 g/day Group 2: 10 g/day	16 weeks	No effect on occurrence, anatomical localization, and degree of pain parameters or headache frequency.
Rist (2021) [75]	Randomized, double-blind, placebo-controlled trial	1032 (65.6 y)	Migraine	2000 IU/day or marine n-3 fatty acid (1 g/d)	5.3 years (median)	No changes in migraine frequency or severity based on self-report
Batcheller (2014) [78]	Prospective	—	Cluster headache	10,000 IU/day	30 days	Decreased frequency, severity, and duration of headache in 80% of patients.

## 5. Vitamin D Profilaxis in Pediatric Headache

There is increasing evidence of a correlation between vitamin D deficiency and pediatric headache [35,36,80,81,82]. However, only a few studies have evaluated the effect of its supplementation in headache prophylaxis in children (Table 2).

Onofri et al. recently administered in an open label study three different compounds to 99 children suffering from a primary headache. Among these compounds, one contained vitamin D, bisglycinate Mg, L-tryptophan, niacin and vitamin B2. All three compounds showed to be effective in reducing the use of attack therapy and migraine disability defined by the formula: n° crisis X pain intensity/month [83]. Cayiri et al., evaluated the effect of vitamin D supplementation in addition to amitriptyline in migraineurs children aged 8–16 years. Patients were divided into four groups based on vitamin D blood level. Groups 1 and 2, with normal vitamin D, received amitriptyline alone and 400 IU/day vitamin D as add-on respectively. Group 3 and 4, with mild and severe vitamin D deficiency, were given 800 and 5000 IU/day vitamin D plus amitriptyline respectively. Vitamin D prophylaxis was revealed superior to amitriptyline alone in the prevention of migraine attacks [84]. In a retrospective study, Kilic et al. analyzed the effect of vitamin D therapy on the frequency, duration, severity of migraine attacks and Pediatric Migraine Disability Assessment (PedMIDAS). In addition to confirming the correlation between more severe migraine and vitamin D deficiency/insufficiency, all endpoints analyzed were significantly improved in treated patients compared with control [36]. In a study published only in abstract form, 37 children with migraine and/or tension-type headaches were treated with a stratified dosage of vitamin D based on the initial level of the vitamin. The study revealed a reduction in headache strength after the supplementation [85].

Even if vitamin D appears effective in reducing headache frequency and severity in the pediatric population, only four studies in the literature have studied this correlation. In addition, some have used vitamin D alone while others together with nutraceutical compounds or prophylactic drugs. It is, therefore, difficult to draw definitive conclusions and further studies are needed to confirm the beneficial effect of vitamin D on pediatric headaches.

## 6. Conclusions

Studies conducted so far on vitamin D seem to point towards a correlation between vitamin D deficiency and increased risk of headache. This finding is confirmed by the possible pathophysiological involvement of vitamin D in the genesis of headache. Moreover, the involvement of vitamin D in both embryogenesis and the adult brain provides the basis for the use of vitamin D in the prophylaxis of headaches in children and adults. The supplementation of vitamin D could not only lead to an improvement of headache frequency and intensity but would also allow reduced use of painkillers, prophylactic drugs and related side effects. Especially at pediatric ages where the use of drugs is greatly burdened by ethical issues and side effects, the possibility of clinical improvement with the use of nutraceuticals such as vitamin D would represent an important therapeutic weapon.

To date, a few studies both in children and adults have evaluated the effect of vitamin D on headache, often associated with nutraceutical formulations or other prophylactic drugs. From the analysis of the data, vitamin D supplementation appears to be related to an improvement in symptoms. However, no official guidelines have defined precise methods and dosages of vitamin D supplementation.

Further randomized studies with a common treatment protocol are necessary to draw definitive conclusions and to encourage the use of vitamin D in headache prophylaxis.

## Figures and Tables

**Table 2:** Vitamin D Profilaxis in Pediatric Headache.

Reference	Study Design	Sample Size (Mean Age)	Type of Headache	Dose of Vitamin D Supplementation	Treatment Duration	Results
Onofri (2020) [83]	Open label study	99 (6–17 y)	Migraine and TTH	Patients were administered 3 different compounds, one containing vitamin D	—	Reduction of migraine disability and of the use of attack therapy
Kilic (2019) [36]	Prospective	42 (14 y)	Migraine	2000 UI for 2 months, 600–1000 IU for next 6 months	8 months	Migraine frequency, duration, severity of attacks and PedMIDAS were significantly improved in treated patients compared with control
Cayir (2014) [84]	Prospective	53 (8–16 y)	Migraine	Group1: amitriptylineGroup2: 400 IU + amitriptylineGroup3: 800 IU + amitriptyline Group4: 5000 IU + amitriptyline	6 months	Group 2-3-4 reported a decrese in headache frequency
Potrykus (2013) [85]	Prospective	37	Migraine and TTH	500–1000–2000–5000 IU based on the initial level of the vitamin D	6 months	Reduction in headache strenght at 3 months

## Data Availability

Not applicable.
